# Voltage/Calcium Uncoupling Underlies Sustained Torsade de Pointes Ventricular Tachyarrhythmia in an Experimental Model of Long QT Syndrome

**DOI:** 10.3389/fphys.2021.617847

**Published:** 2021-01-28

**Authors:** Herman D. Himel, Michael Cupelli, Mohamed Boutjdir, Nabil El-Sherif

**Affiliations:** ^1^Veterans Affairs New York Harbor Healthcare System, Brooklyn, NY, United States; ^2^Downstate Medical Center, State University of New York, Brooklyn, NY, United States; ^3^School of Medicine, New York University, New York, NY, United States

**Keywords:** long QT syndrome, Torsade de Pointes tachyarrhythmias, optical mapping, spatiotemporal entropy, voltage/intracellular calcium uncoupling

## Abstract

**Background:**

Clinical experience showed that the majority of Torsade de Pointes (TdP) ventricular tachyarrhythmia (VT) in patients with long QT syndrome (LQTS) are self-terminating (ST), but the few that are non-self-terminating (NST) are potentially fatal. A paramount issue in clinical arrhythmology is to understand the electrophysiological mechanism of ST vs. NST TdP VT.

**Methods:**

We investigated the electrophysiological mechanism of ST vs. NST TdP VT in the guinea pig Anthopleurin-A experimental model of LQTS, a close surrogate model of congenital LQT3. We utilized simultaneous optical recordings of membrane voltage (V_*m*_) and intracellular calcium (Ca_*i*_) and a robust analytical method based on spatiotemporal entropy difference (E_*d*_) to investigate the hypothesis that early V_*m*_/Ca_*i*_ uncoupling during TdP VT can play a primary role in perpetuation of VT episodes.

**Results:**

We analyzed a total of 35 episodes of TdP VT from 14 guinea pig surrogate models of LQTS, including 23 ST and 12 NST VTs. E_*d*_ values for NST VT were significantly higher than E_*d*_ values for ST VT. Analysis of wave front topology during the early phase of ST VT showed the Ca_*i*_ wave front following closely V_*m*_ wave front consistent with a lower degree of E_*d*_. In contrast, NST VT was associated with uncoupling of V_*m*_/Ca_*i*_ wave fronts during the first 2 or 3 cycles of VT associated with early wave break propagation pattern.

**Conclusions:**

Utilizing a robust analytical method we showed that, in comparison to ST TdP VT, NST VT was consistently predated by early uncoupling of V_*m*_/Ca_*i*_ that destabilized wave front propagation and can explain a sustained complex reentrant excitation pattern.

## Introduction

Patients with both congenital and acquired long QT syndrome (LQTS) are under the risk of developing ventricular tachyarrhythmia (VT), sometimes referred to as Torsade de Pointes (TdP) VT (El-Sherif and Turitto, 1999). Clinical experience showed that the majority of TdP VT are self-terminating (ST) but the few that are non-self-terminating (NST) are potentially fatal (El-Sherif and Turitto, 1999). A paramount issue in clinical arrhythmology is to understand the electrophysiological mechanism of ST vs. NST TdP. This is complicated, on one hand, by the different experimental models of LQTS that do not completely recapitulate all aspects of electrophysiology of the human syndrome. On the other hand, there are current limitations of available investigative techniques that can provide viable explanation. A case in point, a recent experimental study in a canine model of acquired LQTS that utilized tridimensional mapping techniques suggested that short-lasting ST TdP VT had a focal mechanism while long lasting NST TdP VT were maintained by reentrant excitation (Vandersickel et al., 2017). However, the results were criticized in an accompanying editorial because of the controversial mapping definition of focal vs. reentrant excitation (Nayyar et al., 2017).

The present study is designed to investigate the electrophysiological mechanism of ST vs. NST TdP VT in the guinea pig Anthopleurin-A (AP-A) experimental model of LQTS, a close surrogate model of congenital LQT3 (El-Sherif et al., 1988). We utilized simultaneous optical recordings of membrane voltage (V_*m*_) and intracellular calcium (Ca_*i*_) and a novel analytical method based on spatiotemporal entropy (Jung et al., 2000; Bub et al., 2005) to quantify differences in V_*m*_/Ca_*i*_ kinetics during ST and NST TdP VT. Our study will show that, in comparison to ST TdP VT, NST TdP VT is consistently predated by early V_*m*_/Ca_*i*_ uncoupling.

## Materials and Methods

### Experimental Procedure

The experimental model is a Dunkin-Hartley Langendorff-perfused guinea pig heart. The protocol was approved by the institutional animal studies subcommittee, and animals were maintained in accordance with *The Guide for the Care and Use of Laboratory Animals* (National Research Council (US) Committee for the Update of the Guide for the Care and Use of Laboratory Animals, 2011 revised 2011) and ARRIVE guidelines. Voltage and calcium transients are captured using a protocol and equipment fully described previously (Choi and Salama, 2000; Lakireddy et al., 2005). Experimental design, data collection, and analysis techniques were performed as described previously (Himel et al., 2009). Briefly, hearts are collected from guinea pigs (female, 350–450 g) after intraperitoneal injection of heparin (200 U/kg) followed by anesthesia with sodium pentobarbital (35 mg/kg). Hearts are Langendorff perfused with a modified Tyrode’s solution (130 NaCl, 25 NaHCO_3_, 1.20 MgSO_4_, 4.0 KCl, 20 dextrose, 1.25 CaCl_2_; at pH 7.4, bubbled with 95% O_2_–5% CO_2_) with a flow rate of 12–16 mL/min, at 70 mm Hg pressure. Hearts are placed in a custom-made chamber and situated against the imaging surface by positioning a Lexar plunge posterior to the imaged plane. The plunger served to seal the chamber, reduce the curvature of the imaged surface, as well as motion artifacts.

Once the heart is stabilized, atrioventricular block (AVB) is produced by localized injection of 5–10 μL of formaldehyde in the region of the AV node. The heart is paced at a fixed CL of 600–1,000 ms with bipolar Ag-AgCl electrodes placed at the right ventricle outflow tract near the anterior interventricular septum. Ventricular stimulation is applied using constant current pulses of 2.5 ms duration at 1.5× diastolic threshold. The heart is then stained with a voltage-sensitive dye (RH237, Invitrogen, 10–20 μL of a 1 mg/ml solution in dimethyl sulfoxide, DMSO) and intracellular calcium indicator (Rhod-2 AM, Invitrogen, 0.2 mg in 0.2 mL DMSO). The voltage-sensitive dye RH237 is injected as a single bolus over a 1 min period, whereas the calcium indicator is slowly injected in five separate boluses over a 10 min period.

### Drug

Each 100 μg of AP-A (Sigma) is dissolved in 1 mL of water and divided in aliquots of 5–10 μg each, then frozen at -20°C. Aliquots were defrosted, as needed, at room temperature on the day of experiment. Because of possible light sensitivity of the neurotoxin, the perfusion bottle is covered in aluminum foil. After control recordings are obtained, AP-A is perfused at a constant rate of 10–20 μL/min and concentration of 4 nM.

### Optical Mapping

Voltage and calcium transients are captured using a protocol and equipment fully described previously (Choi and Salama, 2000; Lakireddy et al., 2005). Excitation light from a tungsten-halogen source is filtered (520 ± 50 nm) and focused on the surface of the heart. Emitted fluorescent light is collected with a camera lens (85 mm, f1:1.4, Nikon) and passed through a dichroic mirror (630 nm, Omega Optical, Brattleboro, VT, United States). Fluorescence images of the heart are then focused on two 16 × 16 element photodiode arrays (C4675-103; Hamamatsu Corp., Bridgewater, NJ, United States). Ca_*i*_ is imaged using fluorescent light collected below 630 nm and passing it through a 585 ± 20 nm interference filter, while V_*m*_ is imaged by taking fluorescence above 630 nm and filtering with a 715 nm long pass filter. The optical apparatus is adjusted so that voltage and calcium transients are imaged from a 16 × 16 mm square area of the left ventricle. Outputs from the photodiode arrays are amplified up to 1,000 times depending on experimental conditions and digitized at 12-bit resolution prior to offline analysis. V_*m*_ and Ca_*i*_ signals are conditioned by first running a 13 ms moving average filter on each trace, followed by normalization of the data from each trace so that fluorescence values fell between 0 and 1. Traces near the edge of the imaging field (outside of a radius of 8 pixels from the center) were discarded to limit effects of noise on subsequent analysis. The data are then mapped to a color scheme by using a false color filter where red maps to high values of fluorescence, green to intermediate values, and blue maps to low values. Activation maps from 16 × 16 pixels are expanded to 256 × 256 pixel images by linear interpolation. A wave break is defined as the point where the activation wave front and the repolarization wave front join together on the map (Himel et al., 2009).

### Entropy Calculations

We employ a coherent cluster analysis (Jung et al., 2000; Bub et al., 2005; Himel et al., 2009) to compare the spatiotemporal dynamics of V_*m*_ and Ca_*i*_. Successive frames of two-dimensional maps (*x*- and *y*-axis) are stacked along the time axis (*z*-axis) to create a spacetime cube. Active sites are determined by binary comparison to a threshold, which was set to equal to 65% of the maximum ΔF (fractional change in fluorescence relative to the diastolic level of the fluorescence baseline). Nearby active sites are joined to form spacetime clusters. Small clusters can be caused by detector noise and are filtered. Additionally, clusters are not included in the analysis if they do not persist for a minimum time of 50 ms or were not larger than an area of 5 pixels at any given instant. Clusters which were larger than a maximum area of 10 pixels at time zero were removed in order to exclude clusters which began before the analysis window. For a graphical explanation, refer to Supplementary Material. Spatiotemporal entropy is calculated using the following equation:

(1) $$E=-\sum_{s}v_{s}\cdot \ln v_{s}$$

where v_*s*_ is the fraction of the total volume of all clusters for a given cluster size s, as defined by Eq. 2.

(2) $$v_{s}=s\cdot n_{s}/V_{T\,o\,t\,a\,l}$$

where s is the cluster size for a cluster s, n_*s*_ is the number of clusters of size s, and V_*To*__*tal*_ is the total volume of all clusters. Simply stated, v_*s*_ is the fractional volume of all clusters of a given cluster size s relative to the volume of all clusters.

### Use of Entropy to Quantify Uncoupling

As previously reported, entropy is calculated separately for V_*m*_ and Ca_*i*_ data sets for each VT episode (Himel et al., 2009). We do not treat entropy values obtained separately for V_*m*_ and Ca_*i*_ as an accurate measure of the entropy of the underlying system. We obtain an entropy difference value E_*d*_ where E_*d*_ = | EV_*m*_ - ECa| (EV_*m*_ and ECa are the entropy values obtained for V_*m*_ and Ca_*i*_ data sets, respectively). We propose that E_*d*_ is a measure of the difference in complexity between V_*m*_ and Ca_*i*_ dynamics in this system.

VT episodes are located by visual inspection of the data. A 500 ms segment starting at the initiation of each VT episode is isolated for analysis. This interval was carefully chosen after analysis of our preliminary data of the average cycle length (CL) of the overall cardiac excitation rate in the composite electrogram of all short and long VTs during the first 500 ms. The average CL was approximately 160 ± 37 ms, which would include no less than the first 2 and no more than the first 3 beats of the VT episode. The starting point of the 500 ms window is chosen to coincide with the site of the earliest activation wave front in the optical field at the initiation of the VT episode (i.e., the earliest premature beat in the mapping field). Continuous E values are calculated every 160 ms, where each E value represents the E over the 500 ms segment.

Non-sustained VT episodes are classified as self-terminating (ST) and sustained VT episodes as non-self-terminating (NST). NST/VT represented the end of the experiment and no cardioversion of the NST/VT was attempted.

### Statistical Analysis

Values are stated as mean ± standard deviation. A 1-way ANOVA is used to determine whether differences existed among groups. The two-tailed, unpaired Student’s *t*-test with Bonferroni correction was used *post-hoc* to determine differences between groups. The test does not assume equal variance. A corrected critical value of 0.0125 corresponded to an alpha value of 0.05, and is considered to be significant. The Lilliefors test for normality was used to verify that each of the groups tested were normally distributed.

## Results

**FIGURE 1:**
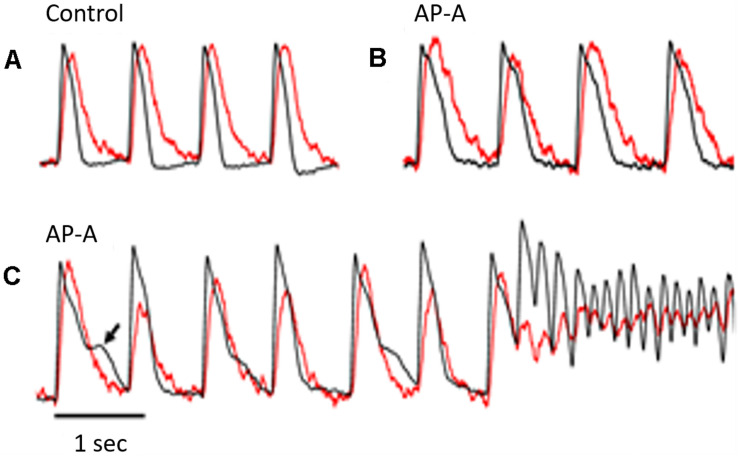
The figure illustrates simultaneous V_*m*_ (in black) and Ca_*i*_ (in red) signals from the same pixel during control **(A)** and following AP-A perfusion **(B,C)**. The heart was paced at a CL of 750 ms. The Ca_*i*_ transient followed the V_*m*_ by approximately 10–15 ms. **(B)** Was obtained 3 min following AP-A perfusion and shows that both V_*m*_ and Ca_*i*_ durations increased by approximately 40–50%. It also shows the development of a moderate degree of alternans of both amplitude and duration of the Ca_*i*_ transient but no detectable change in the V_*m*_ duration. **(C)** Was obtained following 10 min of AP-A perfusion and illustrates the development of significant alternans of the amplitude and duration of the Ca_*i*_ transient. The Ca_*i*_ transient alternans was associated with significant alternans of V_*m*_ duration at plateau level (arrow). A premature beat seemed to arise from the plateau level and initiated a non-self-terminating VT.

**FIGURE 2:**
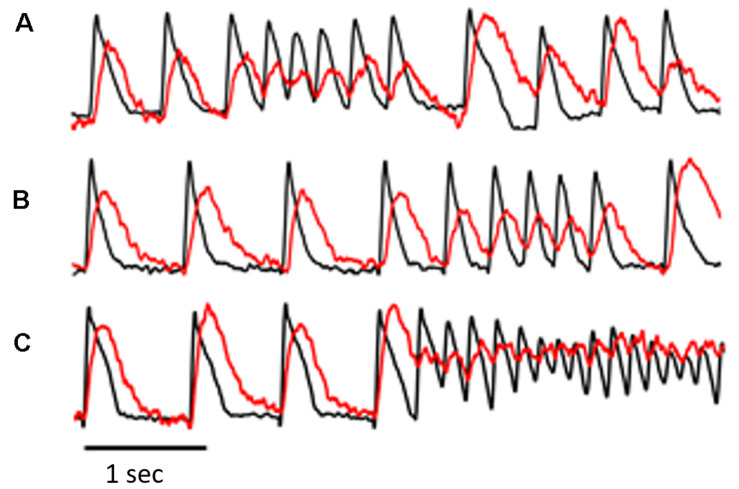
The figure was obtained from a different experiment and illustrates simultaneous V_*m*_ and Ca_*i*_ transient recordings from selected pixels in the epicardial optical window. The recordings in **(A–C)** are consecutive with a few minutes apart. The recordings illustrate a common observation in the AP-A guinea pig surrogate model of LQT3, i.e., the frequent development of ST/VT episodes of variable duration **(A,B)** before the development of NST/VT **(C)**. An interesting observation in this model shown clearly in **(A)** is that the first beat following the end of a ST/VT shows significant prolongation of V_*m*_ duration and a large increase in both amplitude and duration of the Ca_*i*_ transient. Because of the marked prolongation of the Ca_*i*_ transient, the transient of the next paced beat arose before return to baseline of the preceding Ca_*i*_ transient and initiated Ca_*i*_ transient alternans associated with moderate alternation of V_*m*_ duration.

We analyzed 35 episodes of VT from 14 different hearts (23 ST/VTs, and 12 NST/VTs). In 3 hearts NST/VT only occurred (Figure 1). In 3 hearts, one or more ST/VT was only observed, while in 8 hearts one or more ST/VT occurred before the onset of NST/VT (Figure 2). The average duration of ST/VTs (1–10 s) was 2.32 ± 0.81 s (Figure 2). The V_*m*_/Ca_*i*_ uncoupling during the first 500 ms of VT episodes was analyzed. We then analyzed the average CLs of ST/VT and NST/VTs separately. While NST/VTs have slightly shorter CLs of 149 ± 49 ms compared to 161 ± 38 ms for ST/VTs, the difference was not statistically significant.

**FIGURE 3:**
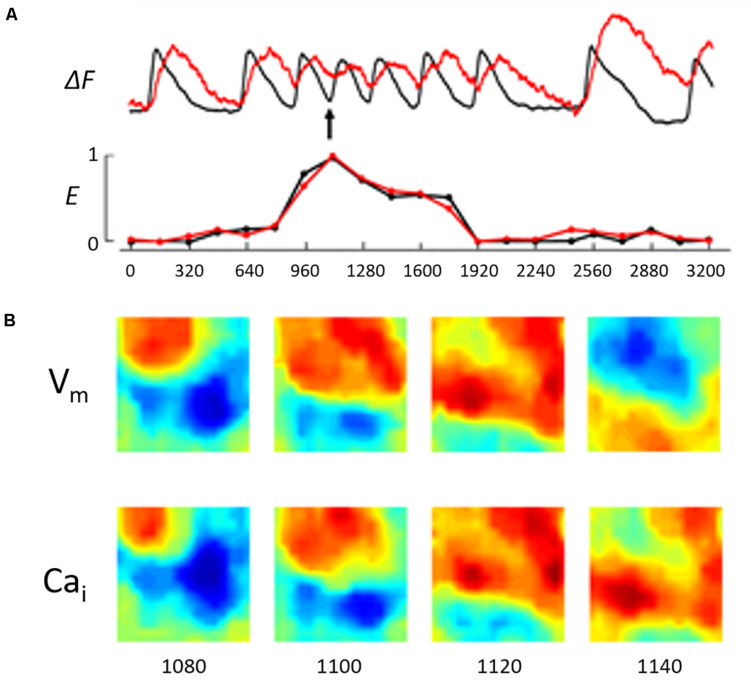
**(A)** Illustrates a running entropy measure during the ST/VT shown in Figure 2B. This was constructed by calculating the entropy value of V_*m*_ and Ca_*i*_ separately at 160 ms intervals. The recording shows the entropy value was close to zero during the basic stimulated beats (first 2 beats and last 2 beats in the recording). During the VT episode the entropy value of both V_*m*_ and Ca_*i*_ increased but remained closely coupled reflecting a low degree of entropy difference. We utilized the difference in entropy values, E_*d*_, as measure of the difference in complexity between V_*m*_ and Ca_*i*_ dynamics in this system. **(B)** Illustrates the wave front topology during the early phase of the ST/VT by showing V_*m*_ and Ca_*i*_ maps in 4 consecutive frames at 20 ms intervals starting 220 ms from the onset of VT (see arrow). The V_*m*_ and Ca_*i*_ activation maps are concordant with the Ca_*i*_ activation wave front closely following the V_*m*_ wave front.

**FIGURE 4:**
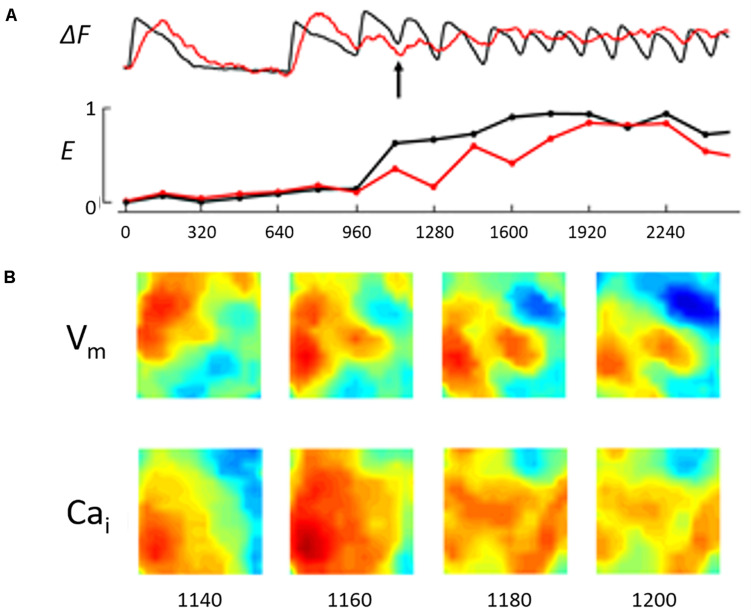
The figure illustrates the V_*m*_/Ca_*i*_ kinetics during the early phase of the NST/VT shown in Figure 1C. **(A)** Illustrates the running entropy during the NST/VT. The entropy values of both V_*m*_ and Ca_*i*_ increased. However, in contrast to the ST/VT, the two entropy values diverged from each other, reflecting a high degree of discordant entropy. **(B)** Illustrates the V_*m*_ and Ca_*i*_ maps in 4 consecutive frames at 20 ms intervals starting at 200 ms from the time of onset of VT (approximately the same as the time frame selected for the ST/VT in Figure 3, see arrow). The V_*m*_ and Ca_*i*_ activation maps show discordant wave front topology with evidence of wave break in both the V_*m*_ and Ca_*i*_ maps.

We calculated E values separately for V_*m*_ and Ca_*i*_, and the difference in E values between them (E_*d*_) was considered a measure of the difference in complexity between V_*m*_ and Ca_*i*_ dynamics. A continuous E measure increased during ST/VT but the E values for V_*m*_ and Ca_*i*_ remained closely coupled (Figure 3A). A continuous measure of E during NST/VT also increased but E values of V_*m*_ and Ca_*i*_ diverged from each other (Figure 4A).

We analyzed the wave front topology during the early phase of ST/VTs and NST/VTs. ST/VTs showed the Ca_*i*_ activation wave front in the optical field following closely the V_*m*_ activation wave front with no evidence of early wave break, consistent with a lower degree of E_*d*_ (Figure 3B). On the other hand, NST/VTs were associated with frequent wave break of both V_*m*_ and Ca_*i*_ wave fronts during the early phase of the VT episode illustrating a higher degree of E_*d*_ (Figure 4B).

**FIGURE 5:**
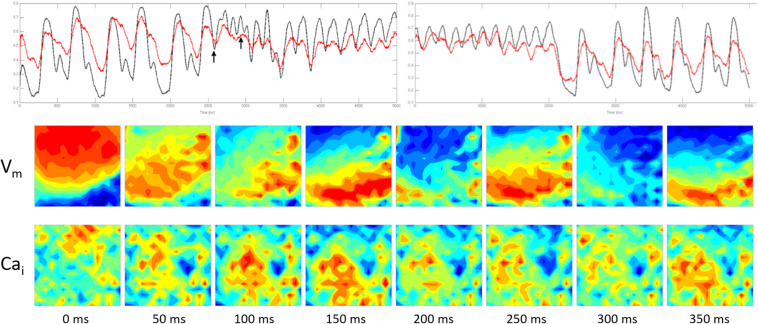
The figure was obtained from a different experiment. Top panel shows simultaneous V_*m*_ (black) and Ca_*i*_ (red) recording of spontaneous ventricular rhythm followed by an early premature beat (first arrow) that initiated a 560 ms episode of fast polymorphic VT at an average CL of 130 ms. The polymorphic VT changed into a monomorphic VT at a CL of 200 ms that lasted for 10 s before it spontaneously terminated. The lower two panels show simultaneous V_*m*_ and Ca_*i*_ wave fronts from the optical field at 50 ms intervals during the first 400 ms of the polymorphic episode (marked by the two arrows). There was no correlation between the V_*m*_ and Ca_*i*_ with frequent wave break of both activation wave fronts reflecting a high degree of E_*d*_. The wave break started immediately after the initiating premature beat in the V_*m*_ recording.

In one experiment a 560 ms episode of fast polymorphic VT at an average CL of 130 ms changed into a monomorphic VT at a CL of 200 ms that lasted for 10 s before it spontaneously terminated. Figure 5 shows simultaneous V_*m*_ and Ca wave fronts from the optical field at 50 ms intervals during the first 400 ms of the polymorphic episode. There was no correlation between the V_*m*_ and Ca_*i*_ with frequent wave break of both activation wave fronts reflecting a high degree of E_*d*_. The wave break started immediately after the initiating premature beat in the V_*m*_ recording.

**FIGURE 6:**
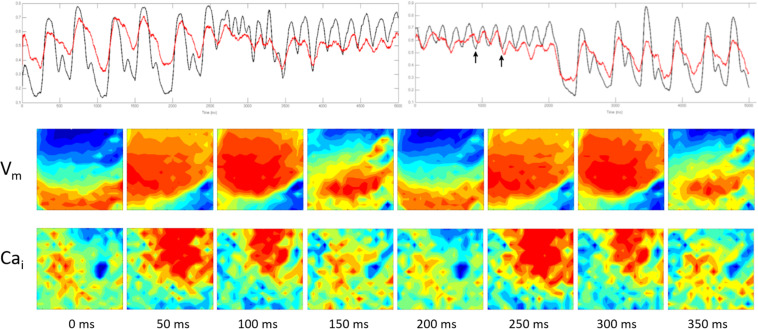
It was obtained from the same experiment shown in Figure 5. The top panel is similar to the top panel shown in Figure 5. The two lower panels show simultaneous V_*m*_ and Ca_*i*_ wave fronts from the optical field covering 400 ms of the monomorphic VT at 50 ms intervals (the section marked by the two arrows). It shows that the Ca_*i*_ activation wave front regularly followed the V_*m*_ wave front with no evidence of wave break reflecting a lower degree of E_*d*_.

Figure 6 shows simultaneous V_*m*_ and Ca_*i*_ wave fronts from the optical field covering 400 ms of the monomorphic VT at 50 ms intervals. It shows that the Ca_*i*_ activation wave front regularly followed the V_*m*_ wave front with no evidence of wave break reflecting a lower degree of E_*d*_.

**FIGURE 7:**
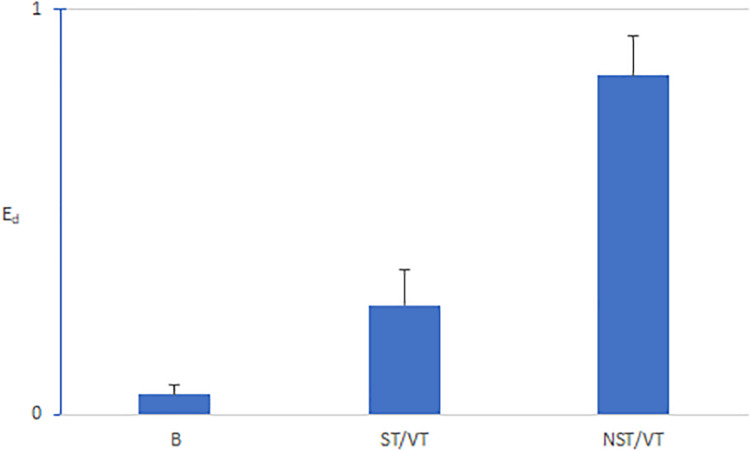
Bar graphs of means and standard deviations of E_*d*_ for the basic rhythm preceding VT episodes (B), ST VTs (S), and NSTVT (NST). The E_*d*_ values during the basic rhythm were consistently close to zero (0.08). The E_*d*_ values for NST/VTs (0.89 ± 0.21) were significantly higher than the E_*d*_ values for ST/VTs (0.33 ± 0.18); (*p* < 0.001).

Figure 7 shows that the average E_*d*_ values during the basic rhythm preceding the onset of VT were consistently close to zero (0.08). The E_*d*_ values for NST/VTs (0.89 ± 0.21) were significantly higher than the E_*d*_ values for ST/VTs (0.33 ± 0.18) (*p* < 0.001).

## Discussion

Our study has shown that, in comparison to ST TdP VT, NST TdP VT is consistently predated by early V_*m*_/Ca_*i*_ uncoupling. In a previous study we utilized spatiotemporal entropy to quantify differences in V_*m*_/Ca_*i*_ kinetics during ST/VT and NST/VT in a Langendorff-perfused guinea pig heart subjected to acute ischemia-reperfusion (Himel et al., 2009). By utilizing simultaneous V_*m*_/Ca_*i*_ optical recordings we showed that early V_*m*_/Ca_*i*_ uncoupling predestinates the duration of VT.

### Experimental Models of LQTS

Both drug-induced and genetically modified animal models of various species have been generated and utilized to investigate the electrophysiological mechanisms of arrhythmogenesis in LQTS. However, species differences, particularly in repolarization currents, prevent these models from recapitulating all aspects of the electrophysiology of the human disease. Genetically modified animal models such as mice and rabbits are commonly used to investigate the arrhythmogenicity of LQTS (Lang et al., 2016).

On the other hand, two dog models of LQTS and TdP have been extensively investigated. One model is the dog with induced complete AVB. Within a few weeks, complete AVB results in hypertrophy and remodeling of the left ventricle associated with prolongation of the QT interval, action potential duration (APD), as well as spatial dispersion of repolarization (DR) (Vos et al., 1998; Kozhevnikov et al., 2002).

The other dog model (El-Sherif et al., 1988) is created by the neurotoxin Anthopleurin-A (AP-A) that accurately reproduces the molecular changes associated with clinical mutations of the Na channel in patients with LQT3. It is worth mentioning that the model was introduced several years before the first description of the clinical LQT3 syndrome (Wang et al., 1995) and has since showed remarkable similarity to the electrophysiological features of the syndrome. In fact, the ionic alterations of delayed Na channel activation by ATX-II (the equivalent of AP-A) by El-Sherif et al. (1992) are almost identical to those reported in the clinical LQT3 patient (Bennett et al., 1995). Later studies have shown that AP-A can also increase overall Na entry via enhanced Na activity (Wasserstrom et al., 1993; Hanck and Sheets, 1995).

In the canine AP-A model of LQTS, analysis of tridimensional activation patterns showed that the initial beat of polymorphic VT consistently arose as focal activity from a subendocardial site, whereas subsequent beats were due to successive subendocardial focal activity, reentrant excitation, or a combination of both mechanisms (El-Sherif et al., 1996, 1997).

### Electrophysiological Mechanisms of the Trigger and Perpetuation of TdP VT in the LQTS

In a previous report by El-Sherif et al. (2019) it was argued that: “Contrary to the established mechanism of the trigger of TdP,” the perpetuation of TdP remains controversial and could be attributed to focal activity, reentrant excitation, or a combination of both mechanisms (Vandersickel et al., 2016, 2017). Both reentrant and focal activity are assumed to be non-stationary. For reentrant excitation, this could be due to a heterogeneity-induced drift of a reentrant circuit (Gray et al., 1995) or a meandering reentrant spiral wave (Winfree, 1995). Alternatively, ectopic beats originating from different locations may explain the perpetuation of TdP.”

It remains an open question why and how ectopic beats emerge and what their relationship to observed EAD activity is. A study by Sato et al. (2009) that combined computational simulation and experimental observations in isolated myocytes showed, “In electrically homogeneous tissue models, chaotic EADs synchronize globally when the tissue is smaller than a critical size. However, when the tissue exceeds the critical size, electronic coupling can no longer globally synchronize EADs, resulting in regions of partial synchronization that shift in time and space. These regional partially synchronized EADs then form premature ventricular complexes that propagate into recovered tissue without EADs thus creating ‘shifting’ foci that resemble polymorphic TdP VT.”

### Measurements of V_*m*_/Ca_*i*_ Uncoupling by Spatiotemporal Entropy vs. Conventional APD Dispersion

Spatiotemporal entropy is a measure that has been applied to quantify wave front fractionation in excitable tissues (Jung et al., 2000). E characterizes wave fronts over a spatiotemporal volume, as opposed to comparing time series on a trace-by-trace basis. E does not reflect APD dispersion; instead, it quantifies multiple fractionated wave fronts in the observational window at a particular point in time. E demonstrates the presence and extent of V_*m*_/Ca_*i*_ wave fractionation, which can only be implied but not demonstrated by previous studies of LQTS that use APD dispersion.

### Ca_*i*_ Transient/V_*m*_ Duration Alternans and TdP

T-wave alternans (TWA) occurs in patients with congenital and acquired forms of LQTS and may precede the onset of TdP (El-Sherif and Turitto, 1999). Although overt TWA in the electrocardiogram is not common, in recent years, digital processing techniques have made it possible to observe more subtle degrees of TWA that were previously undetectable (Verrier et al., 2011). In a previous study, we analyzed the electrophysiological mechanism of the arrhythmogenicity of alternans in the canine AP-A surrogate model of LQTS. We showed that alternans is associated with an increased degree of DR. The present study clearly demonstrates the frequent occurrence of Ca_*i*_ transient alternans and associated V_*m*_ duration alternans. It also demonstrates, for the first time, that alternans preceded the onset of NST/VT.

### ST Monomorphic VT in LQTS

It is commonly forgotten that a minority of NST/VT in patients with LQTS are monomorphic (in simultaneous ECG leads) rather than polymorphic (some, but not all, can qualify as TdP VT) (El-Sherif and Turitto, 1999). In the present study, 2 of 24 episodes of ST/VTs were monomorphic. Similar observations were reported in the canine AP-A surrogate model on LQTS (El-Sherif, 2001). In a classic review of cardiac arrhythmias by Hoffman and Rosen (1981), the authors showed transmembrane action potential (AP) from a canine Purkinje fiber superfused with cesium chloride (a surrogate experimental model of LQT2). The recording illustrates the classic bradycardia-dependent prolongation of APD associated with membrane oscillations on late phase 2/early phase 3 characteristic of EADs. It also showed that complete repolarization of the AP was followed by a subthreshold delayed after depolarization (DAD). The DAD was explained on the basis of increased intracellular Ca^2+^ associated with prolonged APD triggering a transient inward current. This may suggest that some rare monomorphic VTs in LQTS could be secondary to DADs.

### Study Limitations

Our measurements are obtained from a small two-dimensional window of a considerably larger cardiac tissue volume. Therefore, it is impossible to fully track the origin and fate of most wave fronts propagating through the tissue. This limitation also applies to entropy calculations.

## Conclusion

We have shown, for the first time, that differences in wave front topology of V_*m*_ and Ca_*i*_ during the first 2 or 3 cycles of TdP VT, which reflects a higher degree of spatiotemporal entropy difference, is associated with an NST/VT episodes as compared to ST/VT. We have also demonstrated the rare, but clinically relevant, phenomenon of monomorphic NST/VT, which suggest an important role of DAD activity in VT episodes.

## Data Availability Statement

The raw data supporting the conclusions of this article will be made available by the authors, without undue reservation.

## Ethics Statement

The animal study was reviewed and approved by VA New York Harbor Healthcare System IACUC.

## Author Contributions

NE-S and MB designed the experiment. HH collected the data. HH and MC analyzed the data and performed the computation and generated figures. NE-S provided the clinical interpretation and expertise and drafted the manuscript. NE-S and MC revised the manuscript. All authors provided input on final manuscript.

## Conflict of Interest

The authors declare that the research was conducted in the absence of any commercial or financial relationships that could be construed as a potential conflict of interest.

## Abbreviations

AP: action potential
AP-A: Anthopleurin-A
APD: action potential duration
AV: atrioventricular
AVB: atrioventricular block
Ca_*i*_: intracellular calcium
CL: cycle length
DAD: delayed after depolarization
DR: dispersion of repolarization
E: spatiotemporal entropy
EAD: early after depolarization
E_*d*_: magnitude of difference of spatiotemporal entropy between V_*m*_/Ca_*i*_
LQTS: long QT syndrome
NST: non-self-terminating
ST: self-terminating
TdP: Torsade de Pointes
TWA: T-wave alternans
V_*m*_: transmembrane voltage
VT: ventricular tachyarrhythmia.
