# Supplementary material for: Voltage/Calcium Uncoupling Underlies Sustained Torsade de Pointes Ventricular Tachyarrhythmia in an Experimental Model of Long QT Syndrome
Source: Front Physiol. 2021 Jan 28;12:617847. doi: 10.3389/fphys.2021.617847 (PMC7876465; doi:10.3389/fphys.2021.617847)
Supplement: Supplementary file 1 [file Data_Sheet_1.PDF]

Supplement

Included with permission from:

Himel, H. D., Bub, G., Yue, Y., & El-Sherif, N. (2009). Early voltage/calcium uncoupling predestinates the duration of ventricular tachyarrhythmias during ischemia/reperfusion. Heart Rhythm, 6(9), 1359–1365. doi:10.1016/j.hrthm.2009.06.032

Visual Representation of Spatiotemporal Entropy

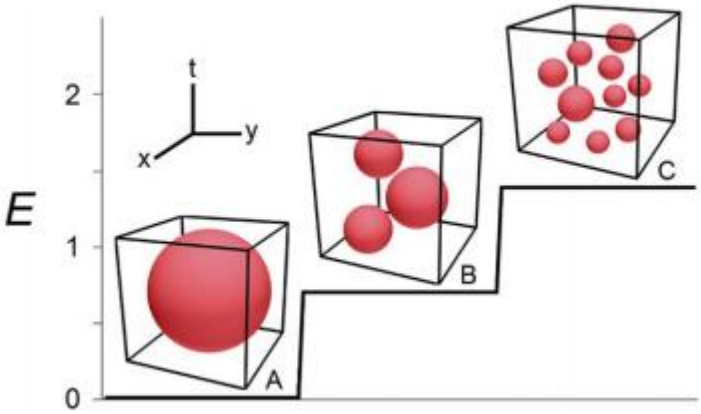

The figure above shows space-time cubes illustrating various levels of entropy. Cube A shows a space-time cube with only 1 cluster comprising 100% of the total volume of all clusters, which gives $v_s = 1$ and $E = -\ln(1) = 0$. Cube B contains 1 cluster comprising 50% of the total volume and 2 others that are 25% of the total volume each. For this case, there are 2 distinct classes of clusters, both of which comprise 50% of the total volume, giving gives $v_s = 0.5$ and $E = -\ln(0.5) = 0.6931$. Cube C contains 4 classes of clusters, including 1 with a volume of 25%, 2 with a volume of 12.5%, 3 with a volume of 8.33%, and 4 with a volume of 6.25%. Thus, each class comprises 25% of the total volume, giving gives $v_s = 0.25$ and $E = -\ln(0.25) = 1.3863$. For the sake of simplicity, classes of clusters in cubes B and C were constructed so that each class comprised an equal proportion of the total cluster volume. Axes for the x-y mapping plane and time are indicated above cube A.
